# Critical fluctuations in epidemic models explain COVID-19 post-lockdown dynamics

**DOI:** 10.1038/s41598-021-93366-7

**Published:** 2021-07-05

**Authors:** Maíra Aguiar, Joseba Bidaurrazaga Van-Dierdonck, Javier Mar, Nicole Cusimano, Damián Knopoff, Vizda Anam, Nico Stollenwerk

**Affiliations:** 1 Basque Center for Applied Mathematics (BCAM), Alameda Mazarredo, 14, 48009 Bilbao, Spain; 2 Dipartimento di Matematica, Università degli Studi di Trento, Via Sommarive, 14-38123 Povo (Trento), Italy; 3 Public Health, Basque Health Department, Rekalde Zumarkalea 39A, 48008 Bilbao, Spain; 4 Osakidetza Basque Health Service, Debagoiena Integrated Healthcare Organisation, Research Unit, Arrasate-Mondragón, Guipúzcoa, Spain; 5 Biodonostia Health Research Institute, Donostia-San Sebastián, Guipúzcoa, Spain; 6 Economic Evaluation Unit, Kronikgune Institute for Health Services Research, Barakaldo, Spain; 7 Ikerbasque, Basque Foundation for Science, Bilbao, Spain

**Keywords:** Applied mathematics, Statistics, Computer modelling, Nonlinear phenomena, Phase transitions and critical phenomena

## Abstract

As the COVID-19 pandemic progressed, research on mathematical modeling became imperative and very influential to understand the epidemiological dynamics of disease spreading. The momentary reproduction ratio *r*(*t*) of an epidemic is used as a public health guiding tool to evaluate the course of the epidemic, with the evolution of *r*(*t*) being the reasoning behind tightening and relaxing control measures over time. Here we investigate critical fluctuations around the epidemiological threshold, resembling new waves, even when the community disease transmission rate $\beta$ is not significantly changing. Without loss of generality, we use simple models that can be treated analytically and results are applied to more complex models describing COVID-19 epidemics. Our analysis shows that, rather than the supercritical regime (infectivity larger than a critical value, $\beta > \beta _c$) leading to new exponential growth of infection, the subcritical regime (infectivity smaller than a critical value, $\beta < \beta _c$) with small import is able to explain the dynamic behaviour of COVID-19 spreading after a lockdown lifting, with $r(t) \approx 1$ hovering around its threshold value.

## Introduction

To assist public health managers and governments during COVID-19 responses, mathematical models were developed within countries’ task forces, first to describe the epidemic in terms of disease spreading and control, giving projections on the national health system necessity during the increased population demand on hospital admissions, for example, based on the available data^1,2^. Valid modeling frameworks continued monitoring disease transmission when the country lockdown was gradually lifted towards the so-called “new normality” and thereafter.

The momentary reproduction ratio *r*(*t*) of an epidemic, defined as the average number of secondary infected cases per infected case in a population in the current state, including both susceptible and non-susceptible hosts, are estimated by simple mathematical models as a function of the community disease transmission rate $\beta$, and controls the transition between a subcritical regime (below the epidemic threshold) and a supercritical regime (above the epidemic threshold).

The evolution of *r*(*t*) is the reasoning behind tightening and relaxing control measures over time, with the transition between subcritical threshold regime ($r(t) < 1$) and a supercritical threshold regime ($r(t) > 1$) observed in many European countries during lockdowns implementation and lifting^3^. While in subcritical regimes, an index infected case will cause an outbreak that will die out sooner or later, the supercritical regimes lead to an exponential growth of infection. The super- or subcritical regimes of an outbreak are often not distinguished when close to the epidemic threshold, where large fluctuations are observed. Identifying the outbreak regime is of major importance to understand the course of an epidemic, improving science-driven policies for disease prevention and control programs in the current epidemiological scenario.

In a basic Susceptible-Infected-Recovered (SIR) epidemiological models with infectivity $\beta$ and recovery rate $\gamma$ we observe in a susceptible population *N* the extinction or the exponential growth of infections when the community transmission $\beta$ if respectively below or above a certain threshold, the so-called critical infectivity $\beta _c$. These behaviours are explained by the fact that, during a given time period, infected individuals are recovering from infection respectively faster or slower than susceptible individuals are becoming infected.

It is long known that around the epidemiological threshold all realistic stochastic models with extinction versus growth to non-zero infection levels display large fluctuations around the epidemic threshold, resembling second epidemic waves, even when the community disease transmission rate $\beta$ is in the subcritical threshold and not significantly changing. These fluctuations, rather than details of the infection process, dominate the dynamical behaviour of the system and no matter how complex models are, they fall into so-called universality classes with power law behaviour and preserved exponents^4–9^.

In this manuscript we investigate these critical fluctuations’ behaviour using basic epidemiological models for disease spreading dynamics, to explore possible features of the course of COVID-19 epidemic in the Basque Country in summer and autumn 2020, after the first lockdown lifting. To characterize the dynamics of large fluctuations during an epidemic, we first describe the interplay between critical fluctuations and import in simple SIR type models (modeling analyses are shown in full detail in^10^), taking into account previous results on directed percolation^4,5^ and dynamical percolation^6,7^. The results described for a simple SIR model with import are then generalized to the current stochastic SHARUCD modeling framework^1,2,11^ developed within the Basque Modeling Task Force (BMTF). Our analysis is based on the development of severe cases, i.e. hospitalizations, intensive care unit (ICU) admissions and deceased cases, while the variation of total number of positive COVID-19 cases is largely due to changes in testing capacities. With real data supporting the theory, we show that the subcritical regime $\beta < \beta _c$ can explain the dynamic behaviour of COVID-19 epidemic in the Basque country and in many other European regions, after the lockdown was lifted in summer 2020. Our study also demonstrates that the momentary reproduction ratio *r*(*t*) hovers around its threshold value due to the import, which is usually overlooked in the interpretation of *r*(*t*) and its relation to the community spreading $\beta$.

## Results

### The role of import in epidemiological systems close to the epidemic threshold

#### The SIR model with import around the critical threshold

Basic epidemiological systems which capture many important issues of disease spreading dynamics can be phrased as SIR-type models, with a population of *N* individuals divided into susceptible individuals *S* to an infectious disease under consideration, infected individuals *I* which are capable of infecting susceptible individuals, and recovered individuals *R* which are either immune against the disease during their entire life, or return to become susceptible again, after a gradual waning immunity period.

In the studied population *N*, besides considering that infected individuals transmit the disease to a susceptible individuals, which then turns to be infected as well, we also consider an import factor which refers to the possibility of susceptible individuals inside the studied population becoming infected by an undetected infection chain started outside the studied population. The imported case is most likely a mobile asymptomatic infected individual, either a foreigner visiting the region or a local returning to the country without being detected by the current testing strategy, similarly of what one expects when country lockdowns are completely lifted and mobility has returned to normal.

**Figure 1:**
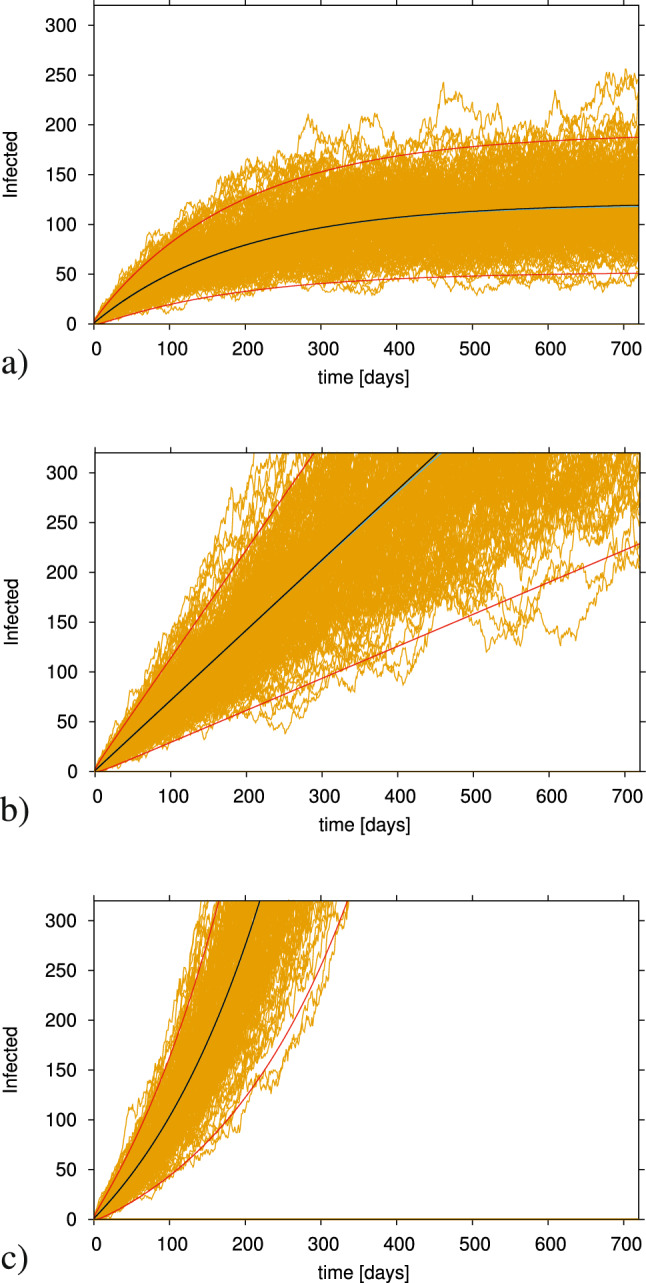
Stochastic realizations, in yellow, and the analytic mean field solution, blue line, for infected in the SIR model. The mean of these stochastic realizations is shown in black and it is in agreement with the mean field solution. With $N=2\cdot 10^6$, $\gamma = 0.05 \; d^{-1}$ and import $\varrho = e^{-12}$, infection rate $\beta$ varies from sub - to super-critical regimes. In (**a**) $\beta =0.9 \cdot \gamma$, in (**b**) $\beta = \beta _c = \gamma$ and in c) $\beta =1.1\cdot \gamma$.

The qualitative behaviour of the stochastic SIR type model with import is shown in Fig. 1 where we investigate the transition between a subcritical epidemic threshold regime and a supercritical threshold regime. We show the stochastic realizations for infected cases in yellow, the mean field solution (in blue) agreeing well with the mean of the stochastic realizations in black, and the variance dynamics of two standard deviations in red above and below the mean curve, which covers roughly 95% in a Gaussian distribution.

Using the basic SIR epidemiological model, we investigate first the case of still controlled community transmission ${\varvec{\beta }} < {\varvec{\beta }} _c$, i.e the subcritical regime. Here, a single index case can generate more new cases before recovering, but on average the newly infected also recover faster than they generate new cases, so that the system would end up with no infected left, after a smaller or larger outbreak.

Nevertheless, with a small import $\varrho$, new index cases can enter the system and cause again similar new isolated outbreaks, no matter how large the import of index cases is. Hence the mean number of infected $\langle I\rangle$ tends not to converge to zero but to a non-trivial endemic state $\langle I\rangle ^*$ which in its simplest form is proportional to the import $\varrho$ and inversely proportional to distance to criticality, namely

1 $$\begin{aligned} \langle I\rangle ^* = \varrho \cdot \frac{\beta }{\beta _c - \beta } \cdot N \end{aligned}$$.

The effect of this scenario is shown in Fig. 1a where we observe, within 200 stochastic realizations (yellow curves), many small outbreaks and occasionally large excursions of the stochastic realizations exceeding occasionally two standard deviations above the mean, even though the system is still in the subcritical regime. The closer to the the critical threshold, ${\varvec{\beta }} \lesssim {\varvec{\beta }} _{\varvec{c}}$, the larger are the observed fluctuations of infected cases.

The limiting case of community spreading at criticality ${\varvec{\beta }} = {\varvec{\beta }} _{\varvec{c}}$ is shown in Fig. 1b, exhibiting linear growth of infections whereas an exponential increase of cases can be only observed for supercritical community spreading ${\varvec{\beta }} > {\varvec{\beta }} _{\varvec{c}}$ (Fig. 1c). In the later case, large fluctuations going below two standard deviations from the mean, indicate that the system is still close to the critical threshold.

#### Subcritical fluctuations and momentary reproduction ratio

**Figure 2:**
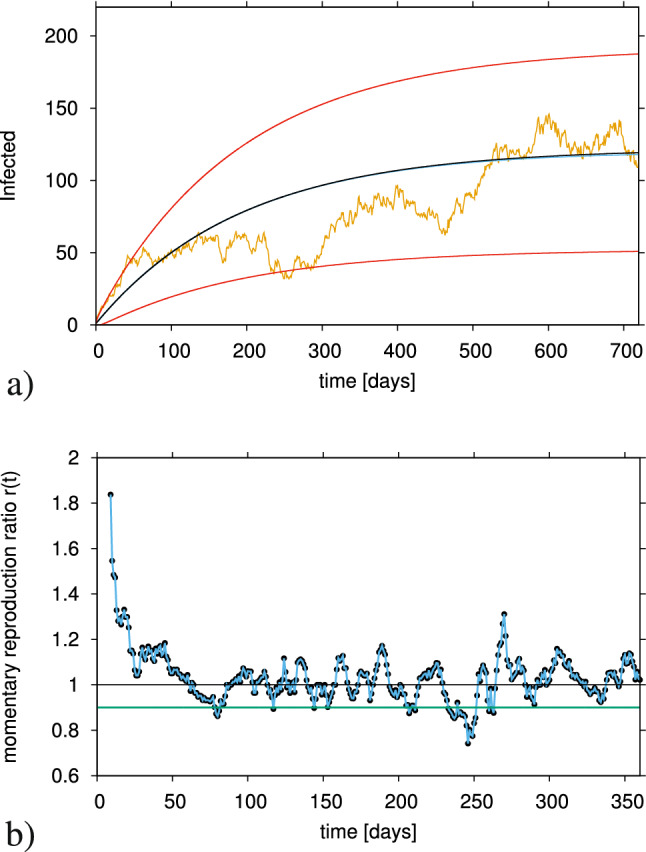
(**a**) Single stochastic realization of the subcritical SIR model with import and constant infection rate $\beta < \beta _c$. Here $\beta = 0.9 \cdot \gamma$, and the other parameters are as in Fig. 1b The momentary reproduction ratio *r*(*t*) calculated from the stochastic realization in Fig. 2a, hovering around the epidemiological threshold of $r_c =1$, even though the theoretical value for a simple SIR model without import is the fixed quantity $r=\beta /\gamma = 0.9 <1$ (green line).

For better orientation we show in Fig. 2a a single stochastic realization of infected cases from the ensamble of stochastic realizations shwon in Fig. 1a. The mean and two standard deviation lines are also shown for orientation.

The present realization shown in Fig. 2a is not atypical since it lies most of the time in the 95% confidence interval and does not even show the often observed large deviations seen in Fig. 1a. However, here we can see that the stochastic realization is oscillating, occasionally in the upper level and occasionally in the lower level the $95\%$ confidence interval.

The data set from this example of an SIR model with import in the subcritical regime is then used to analyze the momentary reproduction ratio *r*(*t*) as described in detail in^3^. The momentary reproduction ratio *r*(*t*) of an epidemic is often used as a public health guiding tool to evaluate the course of an epidemic, assuming that the threshold behaviour of *r*(*t*) reflects that of the threshold behaviour of community spreading $\beta$. However, those measures are only correlated when import is unimportant, i.e, in the exponential growth phase of an epidemic.

In Fig. 2b we observe the momentary reproduction ratio hovering around the epidemic threshold (of decrease to extinction versus exponential growths), even when the infection rate $\beta$ (in green) is constant below the epidemic threshold and as a result the theoretical value of *r* in a system without import (i.e. $r=\frac{\beta }{\gamma }$). From this observation follows clearly that for any epidemiological system with community spreading just below the critical threshold ($\beta > \beta _c$) it is difficult to estimate the reproduction ratio since this would hover above and below threshold due to purely stochastic fluctuations, which are most pronounced around the critical threshold.

### The SHARUCD model update to include analysis of isolated outbreaks after lockdown lifting in the Basque Country

The stochastic SHARUCD modeling framework developed within the Basque Modeling Task Force (BMTF) is an extension of the basic epidemiological Susceptible-Infected-Recovered (SIR-type) models, with infected individuals now distinguishing between severe/hospitalized cases *H* and mild/asymptomatic *A*, including additional classes for intensive care unit admissions *U* and deceased *D*.

**Figure 3:**
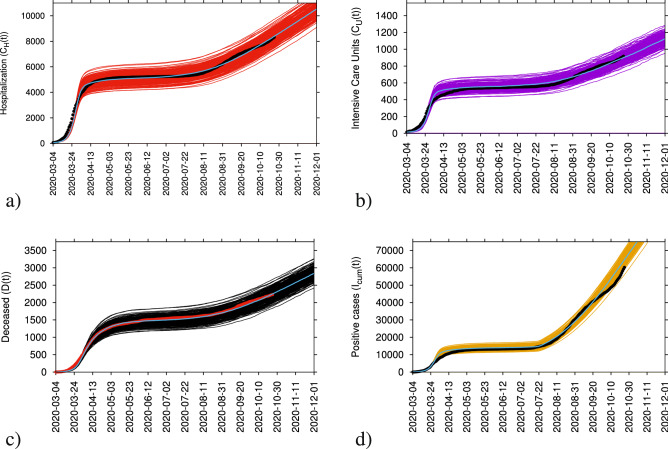
Ensemble of stochastic realizations of the refined SHARUCD-model with import. Starting in March 4, 2020, in (**a**) cumulative hospitalized cases $C_H$(t), in (**b**) cumulative ICU admissions $C_U$(t), in (**c**) cumulative deceases cases *D*(t) and in (**d**) cumulative positive cases $I_{cum}$(t). The mean of 500 stochastic realizations is plotted in blue.

The model was able to describe the COVID-19 epidemic in terms of disease spreading and control, see Fig. 3, and it is currently used to monitor COVID-19 transmission in the Basque Country, see Fig. 3a for hospitalizations, Fig. 3b for ICU admissions, Fig. 3c for deceased cases and Fig. 3d for detected positive cases. Note that as testing capacity increased, the detection rate was adjusted. The system is able to provide accurate projections on the national health system’s necessities during the first wave of the pandemic and beyond, and the complete analysis of the modeling framework is described in detail in^1,2^).

The SHARUCD model was refined to analyze isolated outbreaks, including now import to asymptomatic infection, after lifting lockdown restrictions, and increased detection of asymptomatic due to increasing testing capacity. The deterministic approach described below is obtained via the mean field approximation of the stochastic system, see^1,3,11^. The dynamical system is given by

2 $$\begin{aligned} \frac{d}{dt} S & {} = -\beta \frac{S}{N} (H+\phi A+ (1-\eta )\varrho N) \nonumber \\ \frac{d}{dt} H & {}= \eta (1-\nu ) \beta \frac{S}{N} (H+\phi A)-(\gamma + \mu )H \nonumber \\ \frac{d}{dt} A & {}= (1-\eta ) \beta \frac{S}{N} (H+\phi A +\varrho N)-\gamma A \nonumber \\ \frac{d}{dt} R& {}= \gamma (H+U+A)\nonumber \\ \frac{d}{dt} U& {}= \nu \eta \beta \frac{S}{N} (H+\phi A) - (\gamma +\mu ) U \nonumber \\ \frac{d}{dt} C_H& {}= \eta (1-\nu ) \beta \frac{S}{N} (H+\phi A ) \nonumber \\ \frac{d}{dt} C_A& {}= \xi (1-\eta ) \beta \frac{S}{N} (H+\phi A+\varrho N) \nonumber \\ \frac{d}{dt} C_R& {}= \gamma (H+U+\xi A) \nonumber \\ \frac{d}{dt} C_U& {}= \nu \eta \beta \frac{S}{N} (H+\phi A) \nonumber \\ \frac{d}{dt} D& {}= \mu (H+U) \end{aligned}$$

with time dependent infection rate $\beta$, increased import $\varrho =0,0006$ and increased detection of asymptomatic cases $\xi \in {0.05,0.9}$. Further parameters of the *SHARUCD* models are available in^1,3,11^.

The role of large fluctuations close to the epidemiological threshold in simple models described above is now used to evaluate disease dynamics after the summer lockdown lifting in the Basque Country. Note that the concepts described here still hold even for the period following the second lockdown of November 2020 and further mobility restrictions as of April 2021. Without loss of generality, these results can be applied to more complex models used to describe the COVID-19 epidemics^1–3,11^, where this same dynamical behaviour is observed.

#### COVID-19 epidemiological dynamics in the Basque Country: from the initial phase up to after the summer lockdown lifting

The first cases of COVID-19 in the Basque Country, an autonomous community in northern Spain with 2.2 million inhabitants, were notified on March 4, 2020. A careful data inspection has shown the end of the exponential phase of the epidemic to be around March 26, 2020, allowing us to infer that the partial lockdown was effective and enough to decrease disease transmission in the Basque Country during the first phase of the epidemic. The plan for lifting the restrictions imposed during the state of alarm took place over 4 phases with a “gradual, flexible and adaptive” de-escalation to “a new normality”, starting on May 4, 2020, lasting eight weeks, until the end of June 2020.

The lifting of the lockdown in summer 2020 led to an increase of the infection rate with momentary reproduction ratio hovering around the epidemic threshold. An import factor was included in the model dynamics after the full lockdown lifting in July to describe the opening up of the study area to external influences.

Although this factor was not important during the exponential growths phase in March, 2020, undetected imported infections play a major role during the stochastic pre/post exponential phases, where only small number of infections are detected. The term “import” refers to infected individuals (most likely asymptomatic) coming from outside the studied population (either an infected foreigner visiting the region or an infected Basque returning to the region) that are not detected by the current testing strategy. Note that when the community transmission is under control (social distancing, masks and hygienic measures), the import factor does not contribute significantly to the epidemic, only starting isolated outbreaks.

(variable sizes reflected in the momentary reproduction ratio even under constant community infection rate), but not driving the current epidemic into a new exponential growths phase.

**Figure 4:**
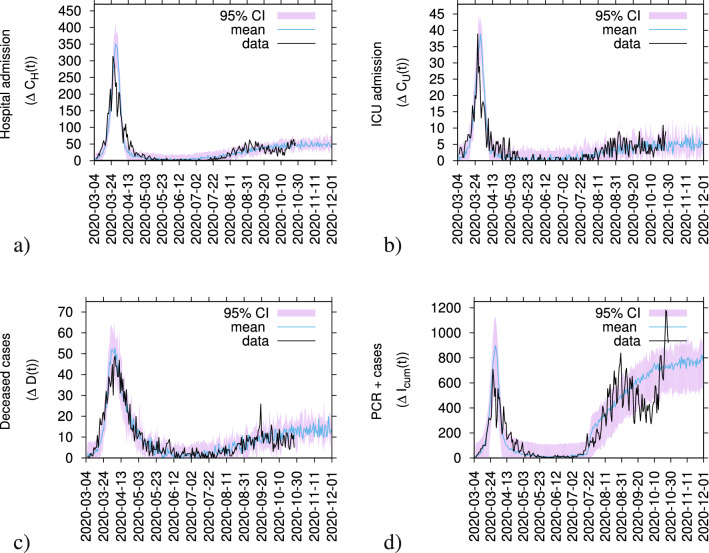
COVID-19 incidences in the Basque Country, from March 4 to October 27, 2020. In (**a**) hospital admissions, in (**b**) Intensive Care Units (ICU) admissions, in (**c**) deceased cases and in (**d**) detected positive cases via PCR tests). Data are plotted as black lines. The mean of 200 stochastic realizations for the SHARUCD model are plotted as a blue line, from March 4 to November 21, 2020, showing a stationarity behavior of the incidences. The $95\%$ confidence intervals are obtained empirically from the stochastic realizations and are plotted as purple shadow.

The observed dynamics in incidences of severe cases, i.e., hospitalized and ICU admissions (Fig. 4a and b respectively) as well as of the deceased cases (Fig. 4c) increased during the initial exponential growth in March 2020 and decreased significantly during the first lockdown, i.e. the controlled phase from April to the end of July 2020.

From the beginning of August onwards, after the complete lockdown lifting, a slight increase in cases is observed, specially the overall detected positive cases (see Fig. 4d), leveling off to stationarity with a number of cases fluctuating around a constant mean. Nevertheless, the number of cases are in the range of the predicted 95% confidence intervals (CI) of the stochastic SHARUCD model, which are themselves subject to fluctuations since the CI are calculated directly from the ensembles of stochastic realizations.

This qualitative behaviour of a slight increase followed by settling into a fluctuating stationary state is observed and holds for a long time, from August to end of October 2020, when the new lockdown was implemented, in very good agreement with the critical behaviour in the simple SIR system under the influence of a conjugate field of import^5^. A final word has to be given to the rather atypical behaviour of the positive detected cases shown in Figs. 3d and 4d. While the predicted qualitative behaviour is consistent with the subcritical regime solution of stationarity, the SHARUCD model has been adjusted to changing testing capacities in order to describe quantitatively the substantial increase of detected positive cases. The observed differences between model predictions and empirical incidence data for the detected positive cases shown in Figs. 3d and 4d can be explained by the significant variance on testing capacity and testing strategies for either population screening or contact tracing over time. Nevertheless, we assume that most of those cases were mild or even asymptomatic cases since this increase did not reflect on notified severe cases, namely hospitalizations, ICUs and deceased.

#### Further data supporting the present analysis: Spanish data and other European countries

**Figure 5:**
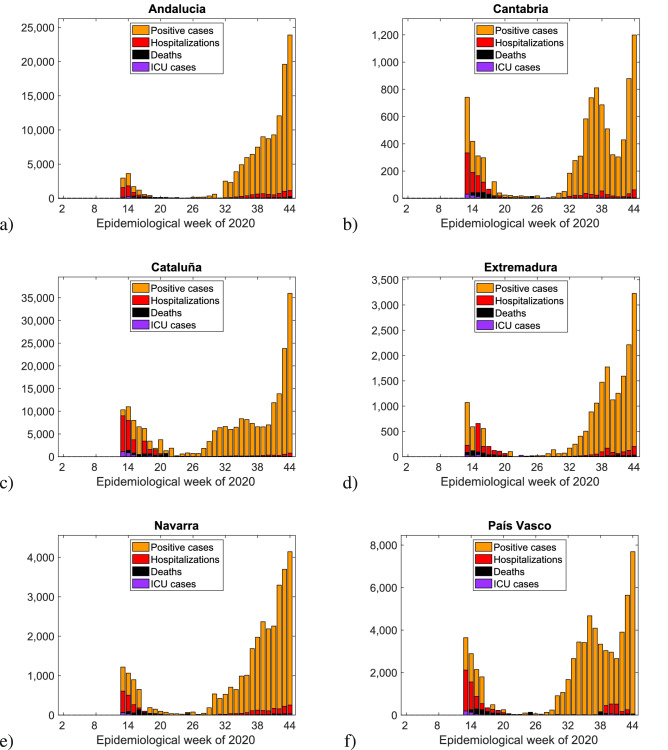
Weekly positive cases, hospitalizations, ICU cases, and deaths in various Spanish autonomous communities until the end of epidemiological week 44 (October 31) of 2020.

**Figure 6:**
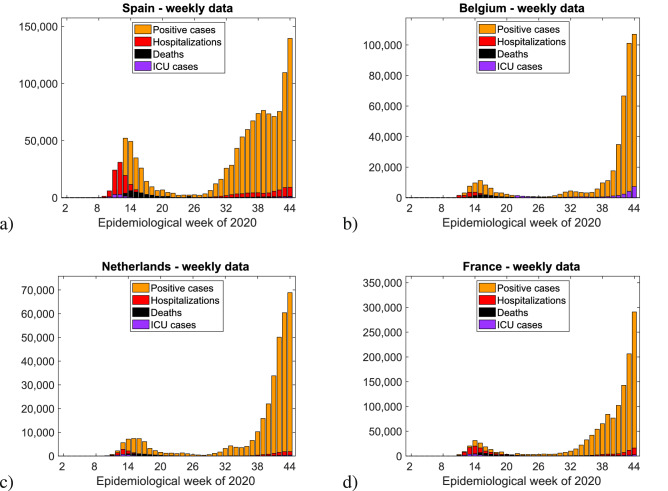
Weekly positive cases, hospitalizations, ICU cases, and deaths in (**a**) Spain, (**b**) Belgium, (**c**) the Netherlands, and (**d**) France until the end of epidemiological week 44 (October 31) of 2020. Only the European Countries where the complete information was available are shown. However, when looking at hospitalizations and deceased cases only, similar behaviour was also found for Germany, Portugal, Sweden, Denmark and Greece.

In Figs. 5 and 6 we plot histograms of weekly new positive cases (yellow), hospital admissions (red), ICU admissions (purple), and deaths (black) associated to COVID-19 for different European regions for which the complete data for all four variables was publicly available.

In Fig. 5 we show the weekly positive cases, hospitalizations, ICU cases, and deaths in various Spanish autonomous communities (in (a) Andalucia, in (b) Cantábria, in (c) Cataluñia, in (d) Extremadura, in (e) Navarra and in (d) País Vasco) until the end of epidemiological week 44 (October 31) of 2020. The cumulative daily values were obtained via the online daily reports given by the Spanish Ministry on Health, Consumer Affairs and Social Welfare^18^. In Fig. 6 we show the weekly positive cases, hospitalizations, ICU cases, and deaths in a) Spain, b) Belgium, c) the Netherlands, and d) France until the end of epidemiological week 44 (October 31) of 2020. Data were collected from the website of the European Centre for Disease Prevention and Control (ECDC)^19^. Hospital and ICU admissions were reported “per 100,000 people” and were here rescaled according to the total population of each corresponding country (as of the end of 2019) as reported in the ECDC data spreadsheets.

The first epidemiological week of 2020 for which data was available varied between countries (hospitalizations: week 5 Spain, week 11 Belgium and France, week 7 Netherlands; ICU admissions: week 5 Spain, week 22 Belgium, week 9 Netherlands, week 11 France) while the last considered week in this work for all countries was week 44 (ending on October 31st 2020).

For the European regions investigated here we observe that although the detected infected cases have increased significantly after the lockdowns were lifted in summer 2020, the severe cases did not follow the same trend but rather remained at much lower level and seemed to approach stationarity, similarly to the described behaviour of subcritical SIR model with import. The increased testing capacities over time were able to detected mainly mild or asymptomatic cases, which were unnoticed during the first epidemic wave in 2020, consequently decreasing case fatality ratios in the various countries, eventually converging to similar magnitudes reflecting biological universality of this measure^20^.

## Discussion

As a continuation of the COVID-19 Basque Modeling Task Force efforts, we investigate the role of critical fluctuations and import in basic epidemiological models on disease spreading dynamics. Without loss of generality, these simple models can be treated analytically and, when considering the mean field approximation of more complex underlying stochastic and eventually spatially extended or generalized network processes, results can be applied to more complex models used to describe the COVID-19 epidemics, where the critical threshold $\beta _c$ is highly non-trivial and not any more simply $\beta _c=\gamma$.

The lifting of the lockdown restrictions in summer 2020 led to an increase of the community transmission $\beta$, with respect to the lockdown phase, but $\beta$ remained below critical threshold due to safety measures still in place, such as mask wearing in public places, avoidance of large crowd gatherings etc. However, lifting of travel restrictions causes the re-opening of systems prone to small import of index cases, which can then spark small or larger isolated outbreaks, as described for the simple SIR system with import, with growth factors and momentary reproduction ratios hovering around the epidemic threshold.

A dynamical behaviour analogously to that observed analytically in the simplest SIR-type models with import close to the critical threshold, but still in the subcritical regime, was found in many European regions, with “severe” disease incidences leveling off to relatively low, but stable values, after the lockdown lifting. With real data supporting the theory, we show that after the lockdown lifting in the summer 2020, community transmission was kept below critical threshold rather than transitioning into the supercritical regime, where a new exponential growth phase would be expected to occur. Sub-threshold community spreading leads to a decline in number of disease cases, but a small import factor is able to ignite isolated outbreaks, occasionally of large sizes, which can lead to a stationary number of new cases in mean field approximation, even when large fluctuations around it are observed. These fluctuations are larger when closer to the epidemic threshold and such increased numbers of cases are treated by many people as “second waves” and inaccurately compared with the first exponential phase in March and April, 2020, where community spreading was definitely well above the critical value. Here, we reserve the term “second wave” to a new exponential growths phase expected in a supercritical community spreading regime, where the number of cases are significantly larger than the actual cases referring to subcritical dynamics with import. Such a new phase of supercritical and hence exponential growth could be expected due to seasonality in respiratory diseases, but observation of more recent data shows that this scenario is likely to have been suppressed by the lockdown measures implemented in November 2020.

Our considerations are based on the observation of empirical data and the analysis of our models assuming that herd immunity caused by natural infection was not reached yet in the Basque Country. Our work differs from other exercises evaluating these critical fluctuations in epidemiological systems as the latter assume low number of susceptible individuals in the system. This assumption would eventually be accurate when large portions of the population will be finally vaccinated.

Here, the assumption of abundance of susceptible individuals gives good results and research questions remain as to which extend vaccines against COVID-19^12–14^ will be needed for effective disease control, with initially reported high vaccine efficacies compared to vaccines for different diseases which were developed over decades^15–17^.

From our analysis we conclude that the way to decrease disease spreading impact in our society, until reaching the herd immunity threshold by vaccination strategies^12,13^, is to implement mild control strategies to keep the system sufficiently below threshold, avoiding large critical fluctuations, but allowing a smooth economic recovery and better planning of necessary long term maintenance activities in many areas of life. The proposed result is able explain the COVID-19 epidemic behaviour in many other European regions and it is of great importance for the upcoming decisions for vaccination programmes implementation and future social distancing restrictions implementation, especially now that “third” and subsequent “new waves” are discussed while the control measures are intermittently relaxed and tightened over and over again.

## Methods

The basis for the present study is the stochastic SIR system, in which susceptible individuals *S* can become infected *I* by meeting already infected individuals with infection rate $\beta$, or by meeting external infected individuals in proportion $\varrho$ (measured as for infected in units of population size *N*). Infected then can became recovered *R* with recovery rate $\gamma$, to become recovered *R*. The dynamics of the recovered *R* can be inferred from the variables *S* and *I* in case of constant population size *N*, hence $R=N-S-I$, and do not need to enter in the probability of state *p*(*S*, *I*, *t*) at any time *t*. Recovered could become susceptible again due to waning immunity or eventually due to new variants (mutating strains of the pathogens). Here we assume this waning immunity rate $\alpha$ as unimportant yet, since complete waning of immunity normally happen after some years. In stochastic processes, infected, susceptible and recovered take integer values.

The dynamics of the stochastic process is then given by the rate of change of probabilities of states

3 $$\begin{aligned} \frac{d}{dt}p(S,I,t)& {}= \beta \frac{S+1}{N} ((I-1)+\varrho N) \; p(S+1,I-1,t) \nonumber \\&+\gamma (I+1) \; p(S,I+1,t) + \alpha (N-(S-1)-I) p(S-1,I,t) \nonumber \\&-\left( \beta \frac{S}{N} (I+\varrho N) +\gamma I + \alpha (N-S-I) \right) \; p(S,I,t) \end{aligned}$$

and can be numerically evaluated e.g. by the Gillespie algorithm to obtain stochastic realizations of the process.

For the dynamics of mean values $\langle I \rangle :=\sum _{I=0}^N I \; p(I,t)$, with similar definitions for $\langle S \rangle$ and $\langle R \rangle$, we then obtain in mean field approximation (see^8^ for further details) the well known ordinary differential equation system of the SIR model with import

4 $$\begin{aligned} \frac{d}{dt} \langle S \rangle& {} = \alpha \langle R \rangle -\beta \frac{\langle S \rangle }{N} (\langle I \rangle +\varrho N) \nonumber \\ \nonumber \\ \frac{d}{dt} \langle I \rangle& {} = \beta \frac{\langle S \rangle }{N} (\langle I \rangle +\varrho N) -\gamma \langle I \rangle \nonumber \\ \frac{d}{dt}\langle R \rangle& {} = \gamma \langle I \rangle -\alpha \langle R \rangle \end{aligned}$$

which is widely studied in mathematical epidemiology, see e.g.^23^ and references therein. In the case of very fast waning immunity $\alpha \rightarrow \infty$ we obtain the SIS epidemic model by bypassing the recovered class. The SIS case is often easier to treat analytically, especially in respect to the here considered import $\varrho$. Spatially extended versions of the stochastic process, including any network structure of connectivities, can easily be formulated as well, see^8,21,22^ and further references there.

At the beginning of an epidemic there is an abundance of susceptible individuals, hence $S/N \approx 1$, and waning immunity can be considered negligible, thus reducing (2) to the following simpler form:

5 $$\begin{aligned} \frac{d}{dt}p(I,t)& {}= \beta (I-1) \; p(I-1,t) + \beta \varrho N \; p(I-1,t) +\gamma (I+1) \; p(I+1,t) \nonumber \\&\quad -\left( \beta I+ \beta \varrho N +\gamma I \right) \; p(I,t) \end{aligned}$$

which can be treated analytically to calculate e.g. mean value $\langle I \rangle$ and variance. The analytic expression for the mean number of infected, when starting exactly with e.g. $I(t_0)$ infected, is given by

6 $$\begin{aligned} \langle I\rangle (t) = I(t_0) \cdot e^{-(\gamma - \beta ) (t-t_0)} + \frac{\beta \varrho N}{\gamma - \beta } \left( 1 - e^{-(\gamma - \beta ) (t-t_0)} \right) \end{aligned}$$

and plotted along the stochastic realizations in the figures discussed in Section 2. For the analytic calculation of the variance, we obtain a lengthy but straightforward analytic expression, see^10^. For the special case when the infection rate $\beta$ is equal to its critical value $\beta _c$ (here coinciding with the recovery rate $\gamma$), we obtain the result

7 $$\begin{aligned} \langle I\rangle _c(t) = I(t_0) + \beta _c \varrho N \cdot (t-t_0) \end{aligned}$$

which is linear in time *t*, as well as in import ratio $\varrho$.

For such spreading into an environment of abundant numbers of susceptible individuals, already without import, one observes the asymptotic behaviour around criticality given by

8 $$\begin{aligned} \langle I \rangle (t,\varepsilon ) \approx t^{{\hat{\eta }}} \cdot F(\varepsilon t^{1/{\hat{\nu }}}) \end{aligned}$$

with critical power law exponents ${\hat{\eta }}$ and ${\hat{\nu }}$, and universal scaling function *F* (see e.g.^7^ and Eq. (15) therein), holding for small $\varepsilon \rightarrow 0$ and long times $t \rightarrow \infty$. The well established values of these critical exponents in 2-dimensional spatially extended stochastic systems ($d=2$) and in mean field approximation (m.f.) are respectively

9 $$\begin{aligned} {\hat{\eta }} _{d=2} = 0.5844, \quad {\hat{\eta }} _{m.f.} = 0 \end{aligned}$$

and

10 $$\begin{aligned} {\hat{\nu }} _{d=2} = 1.5078, \quad {\hat{\nu }} _{m.f.} = 1 \end{aligned}$$

see^7^ and their Figures 16 and 19. For the scaling with import no such results are established, but mean field exponents can be read off from our analytical expression, Eq. (6), giving also here n exponent of 1. The scaling with import, however, has been well studied in the SIS case, and the exponents there are already in mean field approximation more non-trivial, deviating from 0 or 1. For more details on the SIS type model, see^10^.

## Data Availability

Epidemiological data used in this study are provided by the Basque Health Department and the Basque Health Service (Osakidetza), continuously collected with specific inclusion and exclusion criteria, and For the present analysis, the last update was on October 31, 2020. COVID-19 case counts for different European countries are from publicly available datasets: The Spanish Ministry on Health, Consumer Affairs and Social Welfare (e.g., *https* : //*www*.*mscbs*.*gob*.*es*/*profesionales*/*saludPublica*/*ccayes*/*alertasActual*/*nCov*/*home*.*htm*) and the European Centre for Disease Prevention and Control, ECDC COVID-19 data sets (https://www.ecdc.europa.eu/en/covid-19/data).
